# Change in objectively measured physical activity during the transition to adolescence

**DOI:** 10.1136/bjsports-2013-093190

**Published:** 2013-11-22

**Authors:** Kirsten Corder, Stephen J Sharp, Andrew J Atkin, Simon J Griffin, Andrew P Jones, Ulf Ekelund, Esther M F van Sluijs

**Affiliations:** 1 MRC Epidemiology Unit &UKCRC Centre of Diet and Activity Research, University of Cambridge School of Clinical Medicine, Institute of Metabolic Science, Cambridge Biomedical Campus, Cambridge, UK; 2 Norwich Medical School, University of East Anglia, Norwich, UK; 3 Department of Sports Medicine, Norwegian School of Sports Sciences, Ullevål Stadion, Oslo, Norway

**Keywords:** Health Promotion Through Physical Activity, Adolescents, Physical Activity Promotion in Primary Care

## Abstract

**Background:**

Physical activity (PA) declines during adolescence but change in different PA intensities across population subgroups is rarely explored. We describe change in sedentary (SED) time, light (LPA), moderate (MPA) and vigorous PA (VPA) assessed at three time points over 4 years.

**Methods:**

Accelerometer-assessed PA (min) was obtained at baseline (N=2064), 1 and 4 years later among British children (baseline mean±SD 10.2±0.3-year-old; 42.5% male). Change in SED (<100 counts/min (cpm)), LPA (101–1999 cpm), MPA (2000–3999 cpm) and VPA (≥4000 cpm) was studied using three-level (age, individual and school) mixed-effects linear regression including participants with data at ≥2 time points (N=990). Differences in change by sex, home location and weight status were explored with interactions for SED, LPA and moderate and vigorous PA (MVPA).

**Results:**

SED increased by 10.6 (95% CI 9.1 to 12.2) min/day/year. MPA and VPA decreased by 1.4 (1.0 to 1.8) and 1.5 (1.1 to 1.8) min/day/year, respectively. VPA decreased more than MPA as a percentage of the baseline value. MVPA declined more steeply among boys (3.9 (3.0 to 4.8)) versus girls (2.0 (1.2 to 2.7) min/day/year) despite lower MVPA among girls at all ages; rural (4.4 (3.5 to 5.2)) versus urban individuals (1.3 (0.4 to 2.3) min/day/year) and on weekends (6.7 (5.2 to 8.1)) versus weekdays (2.8 (1.9 to 3.7) min/day/year). MVPA was consistently lower among overweight/obese individuals (−17.5 (−3.9 to −2.5) min/day/year).

**Conclusions:**

PA decreases and is replaced by SED during early adolescence in British youth. Results indicate the urgency of PA promotion among all adolescents but especially girls and in rural areas. Increasing VPA and targeting PA promotion during weekends appear important.

## Introduction

Physical activity (PA) in young people has been associated with a reduced risk of obesity^1^ ^2^ metabolic syndrome^3^ ^4^ and beneficial effects on mental health.^5^ Most evidence suggests that PA declines with age throughout adolescence^6^ ^7^ although evidence on the magnitude of the decline is equivocal.^7^ In addition, inactivity may track into adulthood^8^ resulting in a higher risk of health complications later in life.^9^

According to a recent systematic review, PA declines by approximately 7% annually during adolescence.^7^ However, 22 of the 26 studies included used self-reported PA data^7^ which may be susceptible to various forms of bias.^10^ Reported change varied from a decline of 18.8% to an increase of 7.8%^7^ of baseline PA highlighting the inconclusive nature of the evidence. Further detailed examination of the objectively measured PA decline during the transition from childhood to adolescence, using objective measures, is therefore necessary to better establish the nature of this decline to more effectively target PA promotion.

Studies in the recent review mainly examined change among one population group and aside from examining sex differences, few examined change by population subgroup.^7^ Investigating differences between population groups can provide some clarity regarding previous conflicting results and more robust evidence as to the nature and extent of the PA decline. This may aid the identification of priority target groups for PA promotion.

We examine the magnitude of change in objectively measured PA in children over three time points from 9–10 to 13–14 years, and investigate how this change is associated with child sex and weight status, residential setting, parental education and day of the week.

## Methods

### Study design and setting

The Sport, Physical activity and Eating behaviour: Environmental Determinants in Young people (SPEEDY) study is a population-based longitudinal cohort study, investigating factors associated with PA and diet among children attending schools in the county of Norfolk, UK.^11^

### Participant recruitment

Full details on baseline data collection are described elsewhere.^11^ Briefly, primary schools in Norfolk were purposively sampled for urban and rural heterogeneity. From 227 eligible schools (with 5 children ≥12 years), 157 were approached and 92 were recruited. All year 5 children (n=3619) at the 92 schools were invited to participate; 2064 children provided parental consent to participate and were measured at baseline (57% response rate).

### Data collection procedures

Participants were invited to be measured on three separate occasions: baseline (age 9/10 years), +1 (age 10/11 years) and +4 (age 13/14 years).

Baseline data collection occurred between April and July 2007. Researchers visited primary schools to take measurements from 2064 consenting children, administer child questionnaires and fit accelerometers; children returned accelerometers to school 1 week later.

One-year follow-up data collection occurred between April and July 2008 and has been reported previously.^6^ Study information sheets and consent forms were mailed to the homes of all 2064 baseline participants. Those providing parental consent (n=1019) were mailed an accelerometer and instruction sheet and asked to wear the monitor for 1 week and return it by mail.

Four-year data collection occurred between April and August 2011. All participants with valid home addresses after 1-year follow-up (n=1964) were sent information sheets and consent forms. Our original consent did not allow us to trace individual participants so we presented the study at year 9 assemblies at Norfolk secondary schools to encourage any previous participants to participate. Consenting participants were visited at school by researchers to fit accelerometers which were returned to school 1 week later. Owing to low recruitment, an extra invitation letter was sent home at the end of the school term (July 2011) after which an additional 62 participants consented and were assessed by mail during the school holidays as described for 1-year follow-up. In total, 480 participants consented to take part in a 4-year follow-up.

### PA measurement

At all time points PA was assessed using the Actigraph accelerometer (model GT1M). The Actigraph has been shown to accurately assess energy expenditure among European children during free-living conditions.^12^ ^13^ The monitor was set to record the vertical acceleration at 5 s epochs. Children were asked to wear the monitors during waking hours for 7 days and to remove them for water-based activities.

Accelerometry data were analysed using a batch processing programme (MAHUffe: http://www.mrc-epid.cam.ac.uk/Research/PA/Downloads.html) to remove data recorded after 23:00 and before 6:00. Periods of ≥10 min of continuous zeros^14–16^ and days with <500 min of recording^15^ were excluded. All participants with at least 1 day of valid data at ≥2 time points were included in the models.

PA data for each individual were summarised as time spent sedentary (SED), in light (LPA), moderate (MPA), vigorous (VPA) and moderate and vigorous PA (MVPA). Thresholds for defining activity intensities were as follows: SED <100, LPA 101–1999, MPA 2000–3999, VPA ≥4000, MVPA ≥2000 counts/min (cpm). A lower threshold of 2000 (Actigraph) cpm to define MVPA has been used previously in this study^6^ ^11^ and others^16^ and is equivalent to walking at 4 km/h.^12^ Furthermore, this lower threshold for MVPA is very similar to age-specific cut-points commonly used in research in young people.^17^ ^18^ At each time point, mean values (min/day) were derived for all days combined and separately for weekdays and weekends.

To check that results were not solely due to differences in accelerometer wear time at the different measurements, analyses were repeated with accelerometer data expressed as a percentage of the day, for example: change in SED=((follow-up SED min/follow-up worn time)×100)−((baseline SED min/baseline worn time)×100) as used previously.^6^

### Exposure variables

Exposures were assessed at baseline. Researchers used standardised protocols to measure children's height and weight. Height was measured to the nearest millimetre (Leicester height measure, Chasmors Ltd, Leicester, UK). A non-segmental bioimpedance scale was used to measure weight (to the nearest 0.1 kg) and impedance in light clothing (Tanita, type TBF-300A, Tokyo, Japan). Height and weight were used to calculate body mass index (BMI, kg/m^2^). Weight status was derived using sex-dependent and age-dependent cut-points.^19^ Previously validated and published equations were used to calculate body fat percentage (BF%).^20^ Age and gender were self-reported.

The main caregiver self-reported their age at leaving full-time education. Responses were dichotomised into ≤16 and >16 years of age and used as a proxy for socioeconomic status.

Home postcode was used to determine urban/rural location.^21^ Four density profiles were first derived as ‘city’, ‘town and fringe’, ‘villages’ and ‘hamlets and isolated dwellings’; the latter two categories were combined as ‘rural’.

### Statistical analysis

Differences in baseline characteristics for participants with any valid data for ≥2 time points (n=990) and the remaining baseline sample (n=1074) not providing valid data at either follow-up were tested using t tests or χ^2^ tests. We also compared participant characteristics at each time point to the baseline sample where appropriate.

The mean (SD) change in SED, LPA, MPA, VPA and MVPA between baseline and 4-year follow-up was estimated for 409 participants with valid data at both time points (boys n=189; girls; n=220). Absolute change (min/day) was calculated as follow-up (time 3)−baseline (time 1). Relative change (% baseline value) was calculated as the mean of the individual percentage changes in each outcome, calculated as (((follow-up min/day−baseline min/day)/baseline min/day)×100). The percentage of participants who decreased their PA (of all intensities) between their first and last valid measurements was derived. Paired t tests were used to assess whether each outcome had changed between baseline and 4-year follow-up.

Three level mixed effects linear regression models were used to model each outcome at the three time points: baseline (age 9/10 years), 1 (age 10/11 years) and 4 years (age 13/14 years). The exposure variable was age (years) at each time point. The β coefficients for age therefore represent the per-year of age change in PA. Each PA outcome (SED, LPA, MPA, VPA and MVPA) was modelled separately. Models were adjusted for sex, with time points (level 1) nested within individuals (level 2), nested within school attended at baseline (level 3).

Subsequent models additionally included sex–age interactions for all days combined, weekdays and weekend days separately.

Interaction terms were also used to investigate differences in the effect of age by home location, weight status and parental education. Models including interactions were not fit for MPA and VPA separately as the VPA models did not converge, so MVPA was used instead. Analyses were repeated with outcome variables expressed as a percentage of monitor worn time to account for potential differences in wear time between time points. Sensitivity analyses were performed (1) excluding participants recruited during the school summer holidays, (2) including only participants with ≥3 days of valid data at ≥2 time points, (3) using >600 min to define a valid day of accelerometer measurement, (4) excluding 26 participants who reported moving house. Analyses were performed using Stata V.12.0 (Statacorp, College Station, Texas, USA).

## Results

Of the 2064 baseline participants invited to the 1-year follow-up, 1019 (49.4% of original sample) consented and of these, 954 (46.2%) provided Actigraph data. All 1964 (95.2%) baseline participants with valid contact details after the 1-year follow-up were invited to take part in a 4-year follow-up. Of these, 480 consented and 428 provided Actigraph data.

Table 1 presents baseline characteristics of the 990 participants included and participant characteristics for the sample measured at each time point. Compared to baseline, fewer urban children and more girls took part in the 1-year follow-up. Parental education was higher for participants who took part in the 4-year follow-up than those assessed at baseline. Compared to the 1074 children providing only baseline data, the 990 participants with valid data for ≥2 time points were more likely to be girls, had lower baseline BMI z-score and BF% and were less likely to live in an urban location. No statistically significant differences were observed for baseline SED or PA between these groups.

**Table 1:** Characteristics of the SPEEDY sample at waves 1–3

	Baseline	1-Year follow-up†	4-Year follow-up	≥2 Time points‡
N consented	2064	1019	480	
N with ≥1 day PA data (% of consenting sample)	1978 (95.8)	930 (91.2)	428 (89.2)	990 (48.0% of baseline)
N with ≥3 days PA data (% of consenting sample)	1868 (90.5)	882 (86.6)	386 (80.4)	931 (45.1% of baseline)
Age (years)	10.3 (0.3)	11.2 (0.3)	14.3 (0.3)	10.2 (0.3)^2^
Percentage of female	55.6	58.4^2^	53.5^ns^	57.5^2^
Weight (kg)	36.5 (8.4)		57.1 (12.4)	36.1 (8.4)^ns^
Height (cm)	141.0 (6.7)		165.0 (7.6)	141.0 (6.7)^ns^
BMI z-score	0.3 (1.1)		0.4 (1.2)	0.2 (1.1)^2^
Overweight and obese (%)	23.0		20.6	22.0^3^
Body fat percentage	23.6 (6.7)		21.5 (8.1)	23.3 (6.6)^2^
Parental age at leaving education (≤16 vs >16 years)	55.1	57.8^ns^	56.2^2^	57.5^ns^
Home location (all baseline values; %)				
Urban	39.0	33.6^1^	38.2^ns^	35.1^1^
Town/suburban	28.7	30.9	26.0	30.2
Rural	32.3	35.5	35.8	34.7
Sedentary (min)	455.3 (55.9)	469.8 (54.8)	500.7 (58.6)	456.8 (53.7)^ns^
LPA (min)	181.2 (32.7)	177.1 (31.3)	139.6 (33.5)	181.9 (32.50)^ns^
MPA (min)	48.7 (14.5)	47.5 (13.9)	42.9 (15.9)	48.7 (13.9)^ns^
VPA (min)	6.1 (6.5)	6.1 (5.1)	3.0 (4.2)	6.1 (6.6)^ns^
MVPA (min)	73.9 (25.2)	71.7 (22.9)	62.0 (24.7)	73.8 (24.1)^ns^

Participant characteristics are presented for participants with ≥1 day of physical activity data (mean and SD, unless otherwise stated).

t Tests or χ^2^ tests used to test differences between 1-year and 4-year follow-up and ≥2 waves compared to baseline data where appropriate: ^1^p<0.001; ^2^p<0.05; ^ns^p>0.05.

Descriptive data presented for participants with ≥1 day accelerometer data at each wave.

Baseline values presented for participants who had data for ≥2 waves.

Wave 2 was postal data collection and anthropometry was not measured.

†Anthropometry data was not collected at 1-year follow-up.

‡Baseline data presented.

BMI, body mass index; LPA, light physical activity; MPA, moderate physical activity; MVPA, moderate and vigorous physical activity; PA, physical activity; SPEEDY, Sport, Physical activity and Eating behaviour: Environmental Determinants in Young people; VPA, vigorous physical activity.

Table 2 shows 4-year changes in SED and in the different PA intensities for 409 participants with data at both baseline and 4-year follow-up. Relative SED increased by 10.2±17.0% of the baseline value whereas LPA and MVPA declined by 22.4±18.4% and 10.3±41.5% of baseline values, respectively. When examined separately, VPA declined by 11.0±76.3% and MPA by 7.0±39.1% of baseline values.

**Table 2:** Unadjusted change between baseline (age 9/10 years) and 4 years (age 13/14 years) in sedentary time, LPA, MPA, VPA and MVPA over 4 years for participants with data at SPEEDY-1 and SPEEDY-3 (n=409; boys n=189; girls; n=220)

	Baseline (min/day)	Change (min/day)	p Value†	Change (% baseline value)	Percentage of who decreased‡	Baseline (% worn time)	Change (% worn time)	p Value¶
Sedentary								
** **All	460.0±53.8	41.1±71.5*	0.260	10.2±17.0	16.9	64.2±5.9	7.2±6.6§	0.98
Boys	451.3±53.3	45.2±73.5*		11.2±17.4		62.8±6.2	7.2±6.1§	
Girls	467.4±53.3	37.4±67.8*		9.3±16.6		65.3±5.4	7.2±5.8§	
LPA								
All	182.4±31.8	−42.9±35.7*	0.01	−22.4±18.4	69.6	25.4±3.6	−5.6±4.3§	0.01
Boys	184.1±32.2	−38.1±36.7*		−19.4±19.1		25.6±3.7	−5.0±4.5§	
Girls	181.0±31.6	−47.1±34.3*		−24.9±17.4		25.2±3.6	−6.1±4.1§	
MPA								
All	48.9±13.5	−5.9±18.1*	<0.001	−7.0±39.1	58.5	6.8±1.8	−0.7±2.5§	<0.001
Boys	53.2±13.9	−10.0±18.1*		−14.2±36.9		7.4±1.9	−1.3±2.6§	
Girls	45.3±12.0	−2.4±17.3**		−0.8±40.0		6.3±1.6	−0.2±2.3	
VPA								
All	25.9±14.0	−6.8±16.2*	0.96	−11.0±76.3	59.7	3.6±1.9	−0.9±2.3§	0.95
Boys	30.4±15.6	−6.7±17.2*		−4.9±74.6		4.2±2.1	−0.9±2.4§	
Girls	22.1±11.1	−6.8±15.3*		−16.2±77.5		3.1±1.5	−0.9±2.2§	
MVPA								
All	74.9±24.9	−12.6±29.2*	0.009	−10.3±41.5	59.6	10.4±3.3	−1.6±4.1§	0.007
Boys	83.5±26.8	−16.7±31.3*		−13.3±42.2		11.6±3.6	−2.2±4.4§	
Girls	67.4±20.5	−9.2±26.9*		−7.7±40.8		9.4±2.7	−1.1±3.8§	

Change calculated as follow-up−baseline. Values are mean±SD unless otherwise specified.

*p<0.001

**p=0.045 for overall change in min.

†p Value for change in boys versus girls in min. Percentage baseline value calculated as (follow-up min/day minus baseline min/day)/baseline min/day)×100 Percentage worn time calculated as ((follow-up mins/follow-up worn time)×100) − ((baseline mins/baseline worn time)×100)

‡The percentage of participants who experienced a decrease between their first and last measurements.

§p<0.001 for overall change in percentage of monitor worn time.

¶p Value for change in boys versus girls in percentage of monitor worn time.

LPA, light physical activity; MPA, moderate physical activity; MVPA, moderate and vigorous physical activity; SPEEDY, Sport, Physical activity and Eating behaviour: Environmental Determinants in Young people; VPA, vigorous physical activity.

Table 3 shows the estimated average annual changes in SED, LPA and MVPA. All intensities of PA declined and SED increased. When examined separately, there were also declines in MPA (B (95% CI) −1.4 (−1.8 to −1.0); p<0.001) and VPA (−1.5 (−1.8 to −1.1); p<0.001). PA (LPA, MPA, VPA and MVPA) declined for between 58.5% and 69.6% of participants between their first and last measurements and 83.1% of participants increased their SED during the study (data not shown).

**Table 3:** Mean annual change in sedentary time and in light and moderate and vigorous physical activity (MVPA) over three time points: baseline (age 9/10 years), +1 year (age 10/11 years) and +4 years (age 13/14 years)

	Sedentary (min)			Light intensity PA (min)			MVPA (min)		
Subgroup	B	95% CI		B	95% CI		B	95% CI	
Overall	10.6	9.0	12.2	−9.8	−10.7	−8.9	−2.9	−3.5	−2.3
Sex									
Boys	11.5	9.0	14.0	−8.9	−10.1	−7.8*	−3.9	−4.8	−3.0*
Girls	10.0	8.1	11.9	−10.8	−11.7	−9.9*	−2.0	−2.7	−1.2*
Day of the week									
Weekday	8.3	6.0	10.6*	−9.4	−10.3	−8.5	−2.8	−3.7	−1.9*
Weekend	13.4	10.2	16.5*	−10.5	−11.9	−9.1	−6.7	−8.1	−5.2*
Home location									
Urban	9.7	7.1	12.2	−11.0	−12.4	−9.6*	−1.3	−2.3	−0.4*
Town	9.9	6.6	13.1	†			−2.9	−4.0	−1.9*
Rural	12.1	9.9	14.3	−8.5	−10.1	−6.9*	−4.4	−5.2	−3.5*
Weight status									
Normal weight	11.8	10.1	13.4*	−9.5	−10.5	−8.6*	−3.3	−4.0	−2.5
Overweight or obese	6.9	3.8	10.0*	−10.2	−12.4	−8.1*	−2.1	−3.4	−0.8
Parental education (years)									
≤16	10.5	8.4	12.6	−10.5	−12.0	−9.0	−2.8	−3.8	−1.7
>16	10.7	8.7	12.7	−9.4	−10.3	−8.4	−2.9	−3.6	−2.2

Results are from three level mixed effects linear regression models adjusted for sex and stratified to estimate subgroup effects. Values are mean change (min/day/year) with 95% CIs. Participants with valid data at ≥2 time points (n=990) were included in the models except when run separately for weekdays (n=980) and weekend days (n=815). β Coefficients represent the per year changes in the physical activity outcomes.

*Significant interaction.

†This model did not converge.

MVPA, moderate and vigorous physical activity; PA, physical activity.

**Figure 1:**
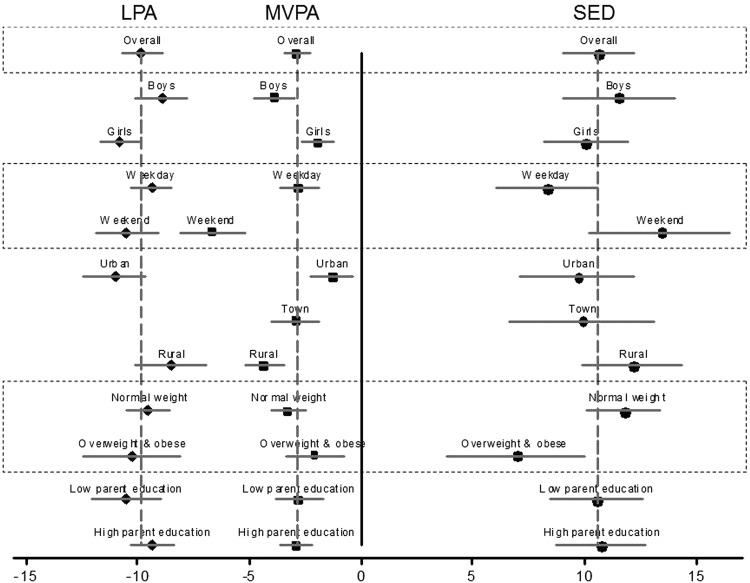
Mean annual change in sedentary time (SED) and in light physical activity (LPA) and moderate and vigorous physical activity (MVPA) over three time points: baseline (age 9/10 years), +1 year (age 10/11 years) and +4 years (age 13/14 years). Results are from three level mixed effects linear regression models adjusted for sex and stratified to estimate subgroup effects. Values are mean change (min/day/year) with 95% CIs. Participants with valid data at ≥2 time points (n=990) were included in the models. The overall B is presented by –- for each group. B for different activity intensities are presented as follows: sedentary (•), LPA (♦) and MVPA (▪). β Coefficients represent the per year changes in the physical activity outcomes.

The effects of age within subgroups defined by biological, demographic and temporal variables are presented in table 3 and displayed in figure 1. The full models including interaction terms are presented in tables 4 and 5. The interactions seen for MVPA are mostly opposite to that for SED, with LPA appearing to fill the gap. Differential declines in MVPA between groups generally resulted in a lessening of the between-group differences observed at baseline. For example, although girls did less MVPA than boys at all time points, sex differences in MVPA decreased over time (table 4). The exception to this was that rural children had lower MVPA than urban children throughout the study and also experienced a greater MVPA decline. Overweight and obese children had lower MVPA throughout the study but the extent of change was not different to normal weight participants (table 5). Overweight and obese children had higher SED throughout the study but normal weight children increased their SED more than the overweight and obese group.

**Table 4:** Average annual change in sedentary time and in light and moderate and vigorous physical activity over three time points: baseline (age 9/10 years), +1 year (age 10/11 years) and +4 years (age 13/14 years)

	Sedentary (min)			Light intensity PA (min)			MVPA (min)		
	β Coefficient	95% CI	p Value	β Coefficient	95% CI	p Value	β Coefficient	95% CI	p Value
All days combined									
Age (years)	10.6	9.1 to 12.2	<0.001	−8.9	−10.1 to −7.8	<0.001	−3.9	−4.8 to −3.0	p<0.001
Sex (reference category: boys)									
Girls	*13.1*	*8.8 to 17.4*	*<0.001*	11.0	−4.7 to 26.7	0.168	**−***37.59*	−*49.9 to −25.3*	** ***p<0.001*
Sex–age interaction (reference category: boys)									
Girls	−1.6	−4.4 to 1.1	0.248	**−***1.7*	−*3.1 to* −*0.2*	*0.022*	*1.89*	*0.8 to 3.0*	** ***p=0.001*
Weekdays									
Age (years)	*7.2*	*5.4 to 8.9*	*<0.001*	**−***8.6*	−*9.8 to* −*7.4*	*<0.001*	**−***2.80*	**−***3.7 to* −*1.9*	*<0.001*
Sex (reference category: boys)									
Girls	*17.2*	*12.9 to 21.5*	*<0.001*	7.71	−8.2 to 23.7	0.343	**−***34.0*	−*46.3 to* −*21.7*	*<0.001*
Sex–age interaction (reference category: boys)									
Girls	−2.1	−5.0 to 0.8	0.152	**−***1.5*	−*3.0 to* −*0.1*	*0.037*	*1.5*	*0.4 to 2.6*	*0.009*
Weekend days									
Age (years)	*13.4*	*10.2 to 16.5*	*<0.001*	**−***10.4*	−*11.8 to* −*9.0*	*<0.001*	**−***6.65*	**−***8.1 to* −*5.2*	*<0.001*
Sex (reference category: boys)									
Girls	*50.6*	*4.4 to 96.8*	*0.032*	−3.5	−7.0 to 0.1	0.052	**−***46.6*	−*67.3 to* −*25.9*	*<0.001*
Sex–age interaction (reference category: boys)									
Girls	**−***4.2*	−*8.4 to* −*0.1*	*0.047*	#			*2.8*	*1.0 to 4.7*	*0.003*

Results are from three level mixed effects linear regression with levels: age, individual and baseline school. Participants with ≥2 time points are included in the models. Results are from models using activity outcomes derived for all days combined (n=990), and separately for weekdays (n=980) and weekend days (n=815). Age–sex interactions were tested for all models. β Coefficients represent the per year changes in the physical activity outcomes.

*Interpretation*: For the all days MVPA model, the average decrease per year of age is 3.9 min/day in boys and 2 min/day (ie, −3.9+1.9) in girls. At age 0, girls do on average 37.6 less min/day than boys, but at age 10 (approximately baseline) this would be (−37.6+10×(1.9)) 18.7 less and at age 14 this would be (−37.6+14×(1.9)) 11.1 less. #This model did not converge.

MVPA, moderate and vigorous physical activity; PA, physical activity.

**Table 5:** Change in physical activity over three time points: baseline (age 9/10 years), +1 year (age 10/11 years) and +4 years (age 13/14 years)

	Sedentary (min)			Light intensity PA (min)			MVPA (min)		
	β Coefficient	95% CI	p Value	β Coefficient	95% CI	p Value	β Coefficient	95% CI	p Value
Home location									
Age (years)	*9.4*	*6.8 to 12.1*	*<0.001*	**−***11.0*	**−***12.4 to* −*9.6*	*<0.001*	**−***1.3*	−*2.3 to 10.4*	*0.006*
Sex (reference category: boys)									
Girls	*13.1*	*8.8 to 17.4*	*<0.001*	**−***7.2*	−*9.8 to* −*4.6*	*<0.001*	**−***17.0*	*−18.9 to −15.2*	*<0.001*
Home location (reference category: urban)									
Town/suburban	−14.7	−57.7 to 28.2	0.501	−6.8	−30.0 to 16.3	0.563	15.7	−0.6 to 32.0	0.059
Rural	−36.6	−76.6 to 3.4	0.073	−21.0	−42.5 to 0.5	0.056	*29.7*	*14.5 to 44.9*	*<0.001*
Age–home location interaction (reference category: urban)									
Town/suburban	0.9	−3.0 to 4.8	0.663	0.90	−1.16 to 2.96	0.392	**−***1.45*	**−***2.89 to* −*0.01*	*0.049*
Rural	3.2	−0.4 to 6.8	0.083	*2.4*	*0.5 to 4.3*	*0.015*	**−***3.0*	−*4.4 to* −*1.7*	*<0.001*
Weight status									
Age (years)	*11.8*	*10.1 to 13.4*	*<0.001*	**−***9.5*	−*10.5 to* −*8.6*	*<0.001*	**−***3.2*	−*3.9 to* −*2.5*	*<0.001*
Sex (reference category: boys)									
Girls	*12.8*	*8.5 to 17.1*	*<0.001*	**−***7.1*	**−***9.7 to −4.5*	*<0.001*	−16.7	−18.5 to 2.3	0.143
Weight status (reference category: normal weight)									
Overweight or obese	*56.3*	*19.6 to 92.9*	*0.003*	14.4	−4.5 to 33.3	0.136	**−***17.5*	*−3.9 to −2.5*	*<0.001*
Age–weight status interaction (reference category: normal weight)									
Overweight or obese	*−4.8*	**−***8.1 to* −*1.4*	*0.005*	−1.2	−3.0 to 0.5	0.157	1.0	−0.4 to 2.3	0.143
Parental education									
Age (years)	*10.6*	*8.4 to 12.9*	*<0.001*	**−***10.5*	**−***11.8 to −9.3*	*<0.001*	*−2.8*	*−3.7 to −1.9*	*<0.001*
Sex (reference category: boys)									
Girls	*13.2*	*8.9 to 17.5*	*<0.001*	**−***7.1*	*−9.6 to −4.5*	*<0.001*	*−17.0*	*−18.9 to −15.2*	*<0.001*
Parental education (reference category: ≤16 years)									
>16 years	9.1	−22.1 to 40.2	0.569	*−16.9*	*−33.0 to −0.9*	*0.039*	−0.9	−13.5 to 11.7	0.885
Age–parental education interaction (reference category: ≤16 years)									
>16 years	−0.1	−2.9 to 2.7	0.942	1.3	−0.2 to 2.7	0.084	−0.1	−1.3 to 1.0	0.812

Results are from three level mixed effects linear regression with levels: age, individual and baseline school. Participants with ≥2 time points (n=990) are included in the models. Results are from models of activity outcomes derived for all days combined, and adjusted for sex. Interactions were tested for all models and presented where significant. β Coefficients represent the per year changes in the physical activity outcomes.

MVPA, moderate and vigorous physical activity; PA, physical activity.

Results were similar when the various sensitivity analyses described in the Statistical analysis section were performed, and when the outcome was expressed as percentage of worn time.

## Discussion

The activity profiles of British children markedly worsen over the transition to adolescence, with over 40 min of PA daily being replaced by SED time over the 4 years of measurement. This is especially concerning considering the potential for this decline to continue throughout later adolescence and the associated adverse health consequences. These results highlight the need to prevent further PA decline among British adolescents.

Our results are in line with a recent review reporting a 7% annual decline in PA throughout adolescence.^7^ Although results support the promotion of all intensities of PA, promotion of VPA may be especially important as it was lowest at baseline, declined rapidly and appears more strongly associated with adiposity and other health outcomes than lower intensity activity.^22^ ^23^

The reduction of 12.6±29.2 min/day in MVPA between 9/10 and 13/14 years could have severe adverse metabolic consequences for adolescent health. A recent meta-analysis examining PA and metabolic outcomes indicated that a 10-min more MVPA is associated with a smaller waist circumference (−0.52 (−0.76, −0.28) cm) and lower fasting insulin (−0.028 (−0.038, −0.017) pmol/L).^4^ Further, we observed an increase in SED of 41.1±71.5 min/day between 9/10 and 13/14 years; an extra hour of SED was associated with higher fasting insulin (0.012 (0.003, 0.02) pmol/L) in the same study.^4^

Differential changes in PA by sex, weekday/weekend and weight status led to smaller between-group differences over time as groups with higher baseline levels experienced steeper changes. This could be due to regression to the mean as those who have lower baseline levels have less scope to change. We previously showed that girls’ MVPA decreased more than boys’ between 10 and 11 years^6^ but the current analyses including data to 14 years, indicated that boys decrease more. This is supported by previous evidence showing a greater decline among younger girls (baseline age 9–12 years), but that boys decrease more than girls during adolescence (baseline age 13–16 years).^7^ However, girls’ PA remained lower than boys’ throughout follow-up and therefore remains an important area of research focus.

Overweight and obese individuals had lower MVPA throughout the study and the extent of change did not differ from normal weight participants, suggesting that overweight and obese individuals should also be a priority for PA promotion. However, weight status may have changed over 4 years, and bidirectional analyses would be necessary to further examine these associations.

The exception to the reduction of between-group differences in PA over time was home location. Compared to urban children, rural children had lower PA levels throughout the study and experienced a steeper PA decline, indicating an increased disparity during the transition to adolescence. There is some indication that cross-sectional correlates of PA may differ according to home location^24^ and PA promotion could emphasise the provision of opportunities for rural children. This observation needs further confirmation in future studies from other populations.

Results highlight the importance of PA promotion and reducing SED among all children as they transition into adolescence but when considering change as well as absolute levels, this seems especially relevant among rural children, girls and those who are overweight or obese. Girls^25^ and overweight individuals^26^ are often highlighted as priority groups for PA promotion, but rural adolescents and weekends have been less frequently identified as important targets. The majority of PA promotion interventions occur in schools^27^ and the observation that PA declines less on weekdays appears to suggest that this may have some benefit. Further development of family and community-based interventions that could better target behaviour during weekends may be especially important.^28^

To our knowledge, this is the first study examining objectively measured PA change in this much detail over 4 years in British adolescents.^7^ Differential follow-up may limit the generalisability of our results, however baseline PA for those included and excluded were similar. Study participants had slightly lower levels of overweight than the general population but the PA decline was similar to previous literature.^7^ Although data was not available from all participants at all time points, the analysis allowed full use of available data and sensitivity analyses including participants with ≥3 days of valid data at ≥2 time points did not differ from the presented results. As results were similar when outcome variables were expressed as a percentage of monitor worn time, we can be confident that these PA changes are not solely due to participants wearing the accelerometers for different lengths of time as they get older. Only 815 participants had ≥1 day of weekend data at ≥2 time points so results for weekend analyses may not be reliable.

## Conclusion

Over 10 min/day of PA every year is replaced by SED time during early adolescence in British youth. Even at baseline, average MVPA was lower than what has been suggested to reduce cardiovascular disease risk^29^ and the extent of the decline potentially puts these adolescents at a greater health risk.^4^ Results indicate the urgency of PA promotion and reducing SED among all children but this seems especially relevant among rural children, girls and those who are overweight or obese. Targeting interventions during weekends and promoting VPA may be especially beneficial. The promotion of at least 10 min of PA daily to replace SED time appears to be a minimal starting point to combat this decline and appears necessary for every year of adolescence.

**What are the new findings?**

- Over 10 min/day of physical activity every year was replaced by sedentary time during early adolescence.
- Physical activity promotion appears important among all children as they transition into adolescence.
- Key intervention targets were identified as girls; children living in rural areas; vigorous activity and weekends.

**How might it impact on clinical practice in the near future?**

- Targeting interventions during weekends and promoting vigorous physical activity may be especially important for adolescent health.
- The promotion of at least 10 min/day of physical activity to replace sedentary time appears to be a minimal starting point to combat the physical activity decline during adolescence.
- Physical activity promotion appears to be important at all ages during the transition from childhood to adolescence.
